# Comparison of tumors with HER2 overexpression versus *HER2* amplification in HER2-positive breast cancer patients

**DOI:** 10.1186/s12885-022-09351-4

**Published:** 2022-03-05

**Authors:** Yoshiya Horimoto, Yumiko Ishizuka, Yuko Ueki, Toru Higuchi, Atsushi Arakawa, Mitsue Saito

**Affiliations:** 1 Department of Breast Oncology, Juntendo University School of Medicine, 2-1-1 Hongo, Bunkyo-ku, 113-0033 Tokyo, Japan; 2 Department of Human Pathology, Juntendo University School of Medicine, 2-1-1 Hongo, Bunkyo-ku, 113-0033 Tokyo, Japan; 3 Department of Breast Oncology, Japanese Red Cross Saitama Hospital, 1-5 Shintoshin, Chuo-ku, 330-8553 Saitama, Japan

**Keywords:** Breast cancer, HER2, Immunohistochemistry, *In situ* hybridization, Trastuzumab

## Abstract

**Background:**

Human epidermal growth factor receptor 2 (HER2)-positive tumors are defined by protein overexpression (3+) or gene amplification using immunohistochemistry (IHC) or fluorescence *in situ* hybridization (FISH), respectively. HER2-positive tumors have historically included both IHC(3+) and IHC(2+, equivocal)/FISH(+) tumors and received the same treatment. Differences in biology between these two tumor types, however, are poorly understood. Considering anti-HER2 drugs bind directly to HER2 protein on the cell surface, we hypothesized anti-HER2 therapies would be less effective in IHC(2+)/FISH(+) tumors than in IHC(3+) tumors, leading to differences in patient outcomes.

**Methods:**

A total of 447 patients with HER2-positive invasive carcinoma who underwent curative surgery were retrospectively investigated. HER2 status was assessed in surgical specimens, except in patients who received neo-adjuvant chemotherapy, where biopsy specimens were employed.

**Results:**

Age, tumor size, lymph node status and ER status were independent factors relating to disease-free-survival, but no difference was observed between IHC(3+) and IHC(2+)/FISH(+) tumors. Kaplan-Meier analysis found patient outcomes did not differ, even after stratifying into those that did (n = 314), or did not (n = 129), receive chemotherapy with anti-HER2 drugs. In 134 patients who received NAC, pathological complete response rates in IHC(3+) and IHC(2+)/FISH(+) tumors were 45% and 21%, respectively. Survival after developing metastasis was significantly shorter in the IHC(2+)/FISH(+) group.

**Conclusions:**

The prognosis of patients with IHC(2+)/FISH(+) tumors did not differ from IHC(3+) tumors. However, the significance of HER2 protein overexpression in relation to treatment response remains unclear and warrants further investigations.

**Supplementary Information:**

The online version contains supplementary material available at 10.1186/s12885-022-09351-4.

## Introduction

Human epidermal growth factor receptor 2 (HER2), a receptor tyrosine-protein kinase, is encoded by the *HER2/neu* gene in humans. Amplification or over-expression of this oncogene plays a crucial role in breast cancer development and progression by inducing downstream pathways, such as PI3K/Akt [1]. Anti-HER2 drugs work by binding to HER2 expressed on the surface of cancer cells. HER2-positive breast cancers previously had poor prognosis [2, 3], but the introduction of trastuzumab, a pioneer anti-HER2 drug, has dramatically improved patient outcomes [4].

Attention is needed, however, in the definition of HER2-positive tumors. HER2 protein expression and *HER2/neu* amplification are clinically assessed with immunohistochemistry (IHC) and fluorescence *in situ* hybridization (FISH), respectively. According to the American Society of Clinical Oncology and the College of American Pathologists (ASCO/CAP) guidelines, a HER2-positive tumor is defined as either IHC(3+) (i.e., overexpressed) or FISH(+) (i.e., amplified) [5]. In practice, however, most cases are first assessed with IHC, and only cases scored as IHC(2+), i.e., equivocal, are assessed with FISH for *HER2/neu* amplification. Generally, HER2 overexpressed IHC(3+) tumors are considered to have *HER2/neu* amplification, with a concordance rate of approximately 90% [6, 7]. In IHC(2+) tumors, FISH is positive in around 10–20% of cases [7–9].

While trastuzumab-based treatments have shown benefit in patients with IHC(3+) or FISH(+) tumors, the definition of HER2-positive tumors differed amongst clinical trials [4, 6, 10–13]. In clinical practice, both IHC(3+) and IHC(2+)/FISH(+) tumors are treated as HER2-positive breast cancer, which is also the case for some new HER2-targeted drugs, such as pertuzumab. Numerous clinical trials have shown both IHC(3+) and IHC(2+)/FISH(+) tumors demonstrated significant benefit from additional trastuzumab, used either alone or in combination with chemotherapeutic drugs [4, 6, 7, 10–14].

Anti-HER2 drugs may be less effective in IHC(2+)/FISH(+) tumors due to less HER2 protein expressed on the cell surface. In a large randomized phase III clinical trial (N9831, n = 1,888) investigating the benefit of additional trastuzumab to adjuvant chemotherapies, patients with IHC-negative and FISH(+) tumors showed no improvement in disease-free-survival with additional trastuzumab, while patients with IHC(3+)/FISH(-) tumors demonstrated disease-free-survival comparable to those with IHC(3+)/FISH(+) tumors, suggesting a key role of protein overexpression [13]. The differences in patient outcomes and efficacy with anti-HER2 therapies between IHC(3+) and IHC(2+)/FISH(+) tumors, however, have not been well studied and are poorly understood.

Multi-gene panel tests have been recently introduced into clinical practice for patients with metastatic breast cancer. These tests evaluate gene status rather than protein expression in the tumor, and are being increasingly used to guide treatment decisions. Indeed, even if HER2 protein expression in the primary tumor is low, anti-HER2 therapy may be offered if the tumor is determined to be HER2-positive by gene panel tests.

The aim of this study was to determine if the therapeutic effects of anti-HER2 therapies and patient outcomes differed between IHC(3+) and IHC(2+)/FISH(+) tumors. We retrospectively investigated patients with HER2-positive invasive breast cancer treated at our hospital, focusing on the differences between patients with IHC(3+) and IHC(2+)/FISH(+) tumors.

## Patients and methods

### Patients

A total of 447 patients with HER2-positive invasive carcinoma underwent curative surgery at our institution from 2010 to 2019. HER2 status was assessed from surgical specimens or, for patients who had received neo-adjuvant chemotherapy (NAC) before surgery, biopsy specimens were assessed to avoid chemotherapy-related effects. Following surgery, standard adjuvant treatments were administered based on tumor characteristics. Details of adjuvant systemic treatments are shown in Additional file 1. Among the 318 patients who received chemotherapy, 171 (54%) patients were given an anthracycline-based regimen; epirubicin plus cyclophosphamide (EC), followed by taxanes (paclitaxel or docetaxel). Another 129 (41%) patients received EC only, while 18 (6%) were given a taxane only. In the 328 patients who received anti-HER2 therapy, trastuzumab was used alone in 289 (88%) patients, while pertuzumab was also used in a combination therapy in 38 (12%) patients. The current retrospective study includes patients who did not receive systemic treatments for some other reasons, such as refusal by the patient, or where the indication for chemotherapy was not clear. This study was performed with approval from the ethics committee of Juntendo University Hospital (H19-0289), and all data were collected after obtaining informed consent from the patients. All data were anonymized before use.

### Pathologic assessment

Pathologic examinations were carried out at Juntendo University Hospital by two experienced pathologists. Tumor grade was judged based on the modified Bloom-Richardson histologic grading system. For patients who received NAC, a pathological complete response (pCR) was defined as the disappearance of invasive nest in the primary breast tumor, i.e., without any lymph node evaluation. Estrogen receptor (ER) and progesterone receptor (PgR) statuses were assessed semi-quantitatively with IHC, and reported as positive when > 1% of cancer cell nuclei showed staining. For the Ki67 labelling index, cells positive for nuclear Ki67 were evaluated semi-quantitatively within a selected hotspot microscopically under high magnification.

The criteria for HER2 assessment were revised slightly by the ASCO/CAP in 2018 [5]. However, this study used the pre-revision criteria [15, 16] since our cases were diagnosed before the 2018 revision. Employing rabbit monoclonal antibody (clone 4B5, Ventana), HER2 protein expression was judged as 0 (negative, no staining observed, or membrane staining in < 10% of tumor cells), 1+ (negative, faint focal membrane staining in > 10% of tumor cells), 2+ (equivocal, weak to moderate staining of the cell membrane in > 10%, or strong staining of the complete membrane in ≤ 10% of tumor cells), and 3+ (positive, strong staining of the complete membrane in > 10% of tumor cells). Patients diagnosed between 2010 and 2012 inclusive, used a 30% cut-off value for 3+, based on ASCO/CAP guidelines during that time [15]. Representative images of IHC are shown in Additional file 2. FISH was conducted using a PathVysion HER2 DNA Probe Kit (Abbott Japan, Tokyo, Japan). *HER2/neu* gene amplification was defined as being present when the FISH ratio was > 2.0. In rare cases, some tumors switched from HER2-negative to HER2-positive on IHC following NAC treatment (e.g., IHC 1 + to 3+). Such cases were excluded from the current study.

### Statistical analysis

Statistical analyses were performed using JMP 11.2.1 statistical software (SAS Institute Inc., Cary, NC). For comparisons of mean values, such as age, examinations of unpaired data were performed employing the two-sided Student’s *t*-test. As a test of independence, the Pearson’s Chi-squared test was used. For evaluation of any independent prognostic effects of the variables, the Cox proportional hazard model was applied with a 95% confidence interval. For continuous variables, mean values were used as the threshold to distinguish between high and low groups. Mean values were 56 (age), 17 mm (pathological tumor size), and 48% (Ki67 labelling index). The full-model analysis selected variables according to their clinical significance, specifically; age, pathological tumor size, lymph node involvement, tumor grade, ER and HER2 status, and administration of chemotherapy. Kaplan-Meier curves were produced and the log-rank test was applied for comparisons of survival between the two populations. A *P*-value < 0.05 was considered statistically significant.

## Results

### Characteristics of patients with HER2-positive tumors

Clinicopathologic features of the 447 patients, including systemic treatments, are shown in Additional file 1. The numbers of IHC(3+) and IHC(2+)/FISH(+) tumors were 398 (89%) and 49 (11%), respectively. Among all 447 patients, 134 received NAC. In total, 318 patients received adjuvant chemotherapies: 54% received an anthracycline-based regimen followed by taxane, 41% received only an anthracycline-based regimen, and 6% received only taxane. HER2-targeted drug(s) were concurrently administered and continued for one year in total. In some NAC cases however, patients started trastuzumab after surgery, due to the drug having just been approved for NAC in Japan at that time (in 2010). Pertuzumab was simultaneously administered with trastuzumab in 12% of patients who received HER2-targeted drugs. Endocrine treatments were given to patients with hormone receptor (HR)-positive tumors according to menstrual status.

We further analyzed these data according to HER2 status (Table 1). In the IHC(2+)/FISH(+) group, significantly more ER and PgR tumors were observed (*P* < 0.001 and *P* = 0.001, respectively), while no differences found between other factors such as tumor grade and the administration of chemotherapy. Reflecting on HR status, adjuvant endocrine therapy was given to more patients with IHC(2+)/FISH(+) tumors (*P* = 0.003).

**Table 1:** Clinicopathological features according to HER2 status (*n* = 447)

		HER2 status				
**Clinicopathological feature**		**IHC(3+)** ^a^		**IHC(2+)FISH(+)** ^a^		***P*** **-value**
n		398		49		
Age (mean)		56.7		54.1		0.227
Tumor size	pTis^b^	54		3		0.059
	pT1	215		23		
	pT2	110		22		
	pT3	19		1		
Lymph node metastasis	Positive	96		14		0.527
	Negative	297		35		
	Not evaluated	5		0		
Histology	NST	350		43		0.970
	Special type	48		6		
Tumor grade^c^	High	121		10		0.130
	Low/intermediate	256		37		
	Not evaluated	21		2		
Ki67 LI (%) (mean)^c^		48.8		42.8		0.133
ER^c^	Positive	245		43		**< 0.001**
	Negative	153		6		
PGR^c^	Positive	178		34		**0.001**
	Negative	220		15		
Chemotherapy	Yes	281^d^		37^e^		0.474
	A + T	155		16		
	A only	111		18		
	T only	15^f^		3		
	No	117		12		
Anti-HER2 therapy	Yes	288		40		0.166
	Tra	255		34		
	Tra + Per	33		6		
	No	110		9		
Endocrine therapy	Yes	237		40		**0.003**
	No	161		9		

*A* anthracycline, *LI* labelling index, *NST* no special type, *Per* pertuzumab, *T* taxane, *Tra* trastuzumab

^a^ Presented as number unless otherwise noted to be mean

^b^ Indicates no remnant invasive disease after NAC

^c^ Assessed on biopsy for neo-adjuvant chemotherapy (NAC) cases

^d,e^ Includes 120 and 14 patients who received NAC, respectively

^f^ Includes one case who received capecitabine as NAC with trastuzumab, but was given taxane after surgery

### Clinicopathological factors relating to NAC

In the 134 patients who received NAC, 57 patients obtained pCR. Clinicopathological features of the 134 patients stratified according to chemo-effect are shown in Table 2. The pCR group exhibited a higher Ki67 labelling index (*P* = 0.009). Significantly more patients who had received a combination of anthracycline and taxane as chemotherapy were observed in the pCR group (*P* = 0.019). No difference in the pCR rate was observed when stratified by ER status. pCR rates in IHC(3+) and IHC(2+)/FISH(+) tumors were 45% (54 of 120 cases) and 21% (3 of 14), respectively, but there was no statistically significant difference (*P* = 0.091).

**Table 2:** Clinicopathological features of neo-adjuvant chemotherapy cases according to chemo-effect (*n *= 134)

		pCR		non-pCR		*P-value*
**Variables**		**n** ^a^	**%** ^b^	**n** ^a^	**%** ^b^	
n		57		77		
Age (mean, range)		52.8	26–81	51.9	26–79	0.665
Histology^c^	NST	53	93	76	99	0.084
	Special type	4	7	1	1	
Tumor grade^c^	High	16	28	24	31	0.980
	Low/intermediate	35	61	52	68	
	Not evaluated	6	11	1	1	
Ki67 LI (%) (mean, range)^c^		61.4	10–90	49.7	5–95	**0.009**
ER^c^	Positive	33	58	46	60	0.830
	Negative	24	42	31	40	
PgR^c^	Positive	18	32	31	40	0.302
	Negative	39	68	46	60	
HER2^c^	3+	54	95	66	86	0.091
	2 + FISH+	3	5	11	14	
Chemotherapy^d^	A + T	56	98	63	82	**0.019**
	A only	0	0	11	14	
	T only	1	2	2	3	
	Others	0	0	1^e^	1	
Anti-HER2 therapy^d^	Tra only	38	67	45	58	0.076
	Tra + Per	8	14	5	6	
	None	11	19	27	35	

*pCR* pathological complete response, *NST* no special type, *LI* labelling index, *A* anthracycline, *T* taxane, *Tra* trastuzumab, *Per* pertuzumab

^a^ Presented as n unless otherwise noted to be mean

^b^ Presented as % unless otherwise noted to be range

^c^ Assessed on biopsy for neo-adjuvant chemotherapy (NAC) cases

^d^ For NAC

^e^ Capecitabe was given with trastuzumab

### Factors relating with patient outcomes

During the mean observation period of 59 months (range, 1-137), 42 patients developed distant metastasis (9.4% of the 447 cases). Fourteen patients (3.1%) died due to breast cancer. Univariate analysis revealed pathological tumor size and lymph node involvement were associated with disease-free-survival (DFS; Table 3). With multivariate analysis, age, tumor size, lymph node status and ER were independent factors (*P* = 0.043, *P* < 0.001, *P* < 0.001, and *P* = 0.012, respectively). In other words, patients that were young, with tumors that were larger, had lymph node metastasis, and/or were ER-negative, had significantly shorter DFS. There was no difference between IHC(3+) and IHC(2+)/FISH(+) tumors.

**Table 3:** Clinicopathological features in relation to disease-free-survival (*n* = 447)

		Univariate				Multivariate	
**Variables**	**HR**	**95%CI**	***P-*** **value**		**HR**	**95%CI**	***P*** **- value**
Age (> 56 vs. ≤ 56)	0.67	0.45–1.24	0.200		0.51	0.25–0.98	**0.043**
Tumor size (> 17 mm vs. ≤ 17 mm)	3.72	1.95–7.56	**< 0.001**		3.24	1.64–6.76	**< 0.001**
Lymph node metastasis (positive vs. negative)	4.93	2.68–9.29	**< 0.001**		4.24	2.23–8.24	**< 0.001**
Histology (NST vs. special type)	0.98	0.42–2.86	0.973				
Tumor grade (high vs. low/intermediate)	0.89	0.43–1.73	0.740		0.88	0.41–1.75	0.725
Ki67 LI (> 48% vs. ≤ 48%)	0.91	0.46–1.81	0.790				
ER (positive vs. negative)	0.58	0.32–1.07	0.082		0.43	0.22–0.83	**0.012**
PgR (positive vs. negative)	0.84	0.45–1.55	0.582				
HER2 (2 + FISH + vs. 3+)	1.08	0.37–2.52	0.866		1.10	0.36–2.75	0.855
Administration of chemotherapy (yes vs. no)	0.92	0.48–1.88	0.814		0.57	0.29–1.21	0.139
Administration of anti-HER2 therapy (yes vs. no)	1.06	0.54–2.27	0.873				

*NST* no special type, *LI* labelling index, *HR* hazard ratio, *CI* confidence interval

**Fig. 1:**
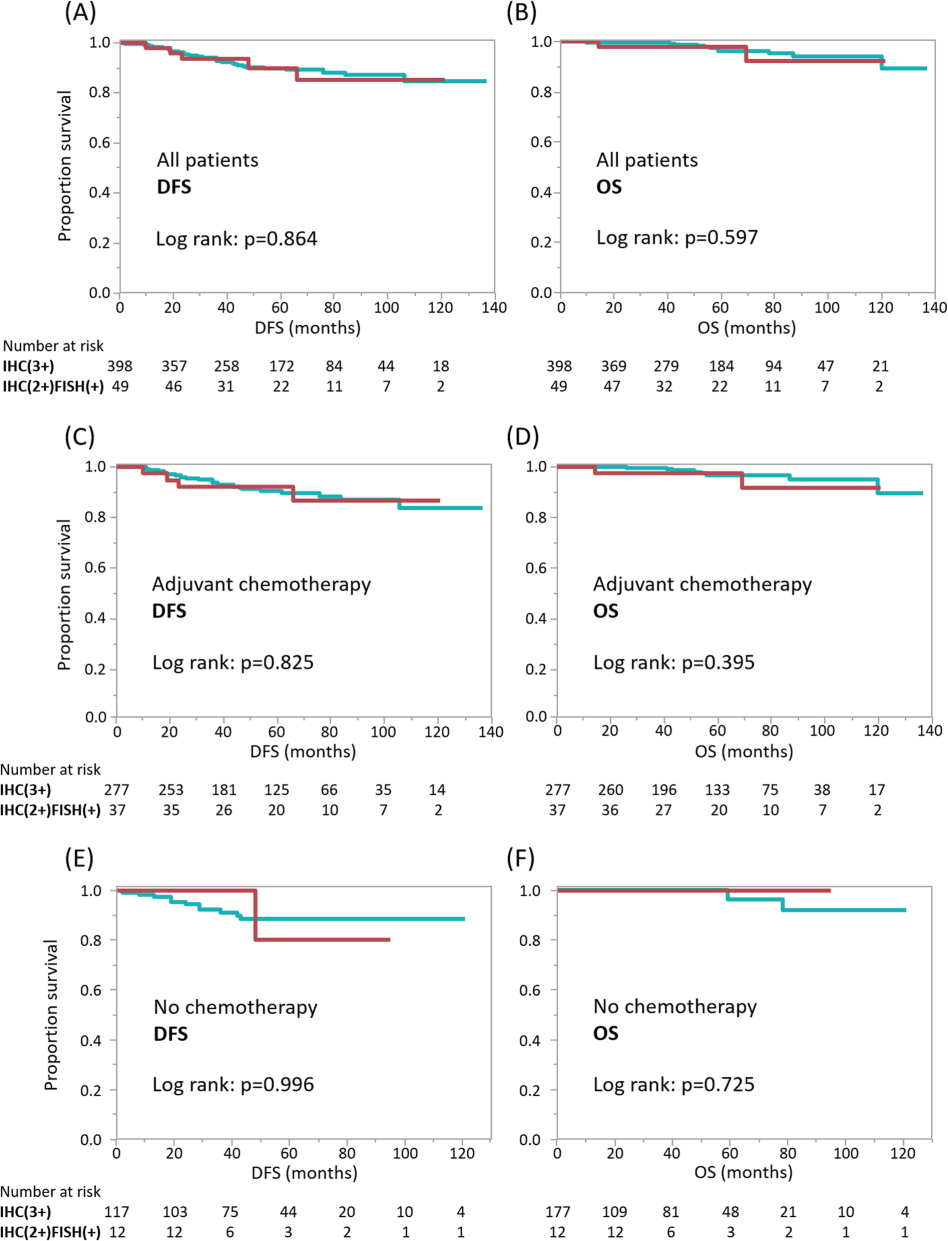
Patient outcomes stratified by HER2 status. ** A**-**B** Kaplan-Meier analyses indicate disease-free-survival (DFS) (**A**) and breast cancer-related overall survival (OS) (**B**) according to HER2 status in all 447 patients. Green and red lines indicate patients with IHC(3+) and IHC(2+)/FISH(+) tumors, respectively. **C**-**D** DFS (**C**) and OS (**D**) in the 314 patients who received adjuvant chemotherapies in combination with anti-HER2 drugs. **E**-**F** DFS (**E**) and OS (**F**) in the 129 patients who did not receive any chemotherapy

Kaplan-Meier analysis was then used to assess differences in patient outcomes between IHC(3+) and IHC(2+)/FISH(+) tumors according to systemic therapies. There was no difference in DFS or breast cancer-related overall survival (OS) between patients with IHC(3+) and IHC(2+)/FISH(+) tumors when evaluating all participants (*n* = 447; Fig. 1 A and B). Similarly, there was no difference in DFS or breast cancer-related OS between patients with IHC(3+) and IHC(2+)/FISH(+) tumors in patients who received a combination of chemotherapeutic and anti-HER2 drugs as standard adjuvant treatment, regardless of whether it was administered pre/post-operatively (*n* = 314; Fig. 1 C and D), or patients who did not receive any chemotherapy (*n* = 129; Fig. 1E F). The four patients who received adjuvant chemotherapy but not anti-HER2 drugs for some reason were excluded from the analysis shown in Fig. 1 C and D. A subset analysis compared DFS in the 133 patients that received NAC, and similarly found no difference in DFS between patients with IHC(3+) and IHC(2+)/FISH(+) tumors (*P* = 0.821; Additional file 3).

Patient outcomes were separately analyzed after stratifying by HR status (Additional file 4). The main results did not change, in that there was no significant difference in DFS or OS between IHC(2+)/FISH(+) and 3 + tumors in all patients or those treated with adjuvant chemotherapy after separating HR-positive and HR-negative tumors. However, in patients with HR-negative tumors, IHC(2+)/FISH(+) tumors had significantly shorter OS than 3 + tumors. The small sample of the IHC(2+)/FISH(+) group (*n* = 4) however, means the significance of this result is inconclusive.

**Fig. 2:**
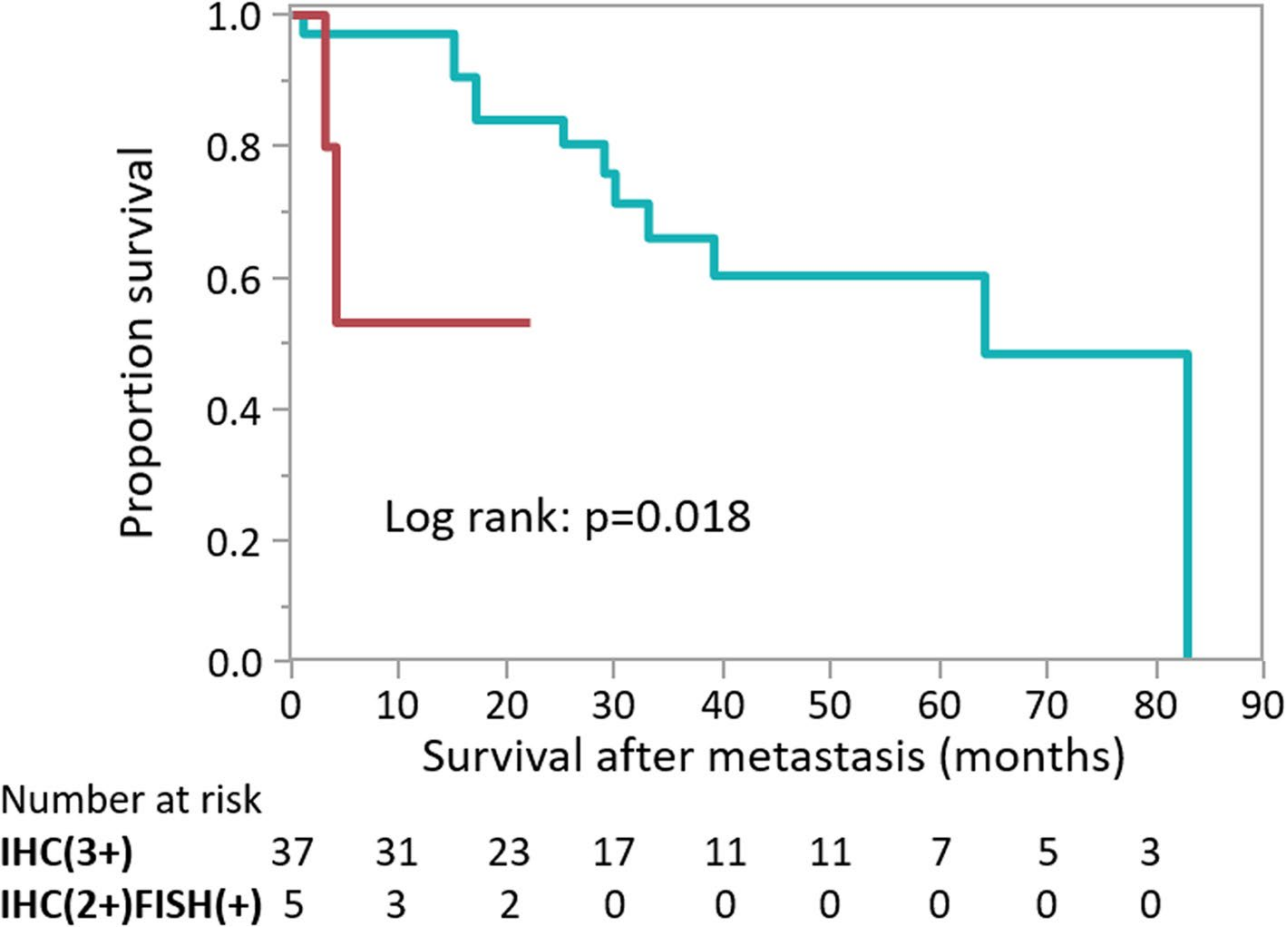
Survival after developing distant metastasis, stratified by HER2 status (*n* = 42). Kaplan-Meier analyses indicate survival after developing distant metastasis. Green and red lines indicate patients with IHC(3+) and IHC(2+)/FISH(+) tumors, respectively

Finally, survival after developing metastasis was analyzed in 47 patients who developed distant metastasis (Fig. 2). The IHC(3+) group (*n* = 37) had a mean survival of 32 months (range, 0-1184), while the IHC(2+)/FISH(+) group (*n* = 5) had significantly shorter survival (mean, 9 months; range, 3–22; *P* = 0.018), although the number of patients in this group was obviously small.

## Discussion

In this study, we found patient outcomes did not differ between breast cancer patients with IHC(3+) and IHC(2+)/FISH(+) tumors. Even after stratifying patients into those that had or had not received adjuvant chemotherapy with anti-HER2 drugs, there was no difference in patient outcomes. To the best of our knowledge, there has been no other report comparing patient outcomes with these two tumors. To determine whether IHC(3+) and IHC(2+)/FISH(+) tumors differ in their intrinsic malignancy, it is necessary to compare patients who did not receive chemotherapy. However, our cohort was retrospectively collected and the application of chemotherapy was not randomized. Moreover, the sample size of the non-chemotherapy group was relatively small. Hence, we were not able to adequately examine this point. It is no longer ethical to employ an arm without anti-HER2 agents in prospective clinical trials. As such, more retrospective observational studies such as the present study need to be collated.

As to the effectiveness of anti-HER2 therapies, in the NAC setting, pCR rates were lower in IHC(2+)/FISH(+) tumors compared with IHC(3+), although the difference did not reach statistical significance. Small sample numbers might have affected the statistical analysis. Meanwhile, patients with IHC(2+)/FISH(+) tumors showed significantly shorter survival after developing distant metastases. Considering anti-HER2 therapies are given to patients with both IHC(3+) and IHC(2+)/FISH(+) tumors, we cannot exclude the possibility that IHC(2+)/FISH(+) tumors do not respond well to anti-HER2 therapies. In the N9831 clinical trial, patients with IHC-negative and FISH(+) tumors had no additional benefit, in terms of prolonging DFS, with receiving trastuzumab with adjuvant chemotherapies [13]. The importance of HER2 protein overexpression merits further investigation. It would be relatively easy to conduct an additional analysis in recent clinical trials that include a variety of anti-HER2 drugs, to evaluate treatment effectiveness in IHC(3+) and IHC(2+)/FISH(+) tumors.

The IHC(2+)/FISH(+) group included significantly more HR-positive tumors. However, this phenomenon is probably caused by selection bias rather than biological difference, as ER-positive tumors were predominant among those in which FISH was examined (data not shown). ER can regulate and activate HER2 signaling [9, 17, 18], thus crosstalk signaling may influence HER2 protein expression in FISH + tumors. However, to test this hypothesis, all FISH + tumors should be examined regardless of IHC results. We could not investigate this issue in the current study as FISH was conducted only in IHC(2+) tumors. Nevertheless, the fact that pCR rate did not differ in relation to ER status indicates chemotherapy with anti-HER2 treatment is effective in ER and HER2-positive tumors, as well as HER2 type (HR-negative and HER2-positive) tumors. Meanwhile, patients with ER-positive tumors had significantly longer DFS. Adjuvant endocrine treatments should, of course, contribute to these patients’ prognoses.

The major limitation of our study was that it was a retrospective observational study, thus systemic treatments, such as chemotherapy and anti-HER2 drugs, were not uniform. Further analysis with a larger sample size is required, especially to further examine patient outcomes of IHC(2+)/FISH(+) and HR-negative tumors, and to compare the effects of treatment after recurrence. In addition, while FISH was only performed in IHC(2+) tumors, it can be assumed that some IHC(0/1+) tumors are also FISH(+) [19]. The biological behavior of FISH(+) tumors with none/little HER2 protein should be thoroughly assessed in the near future, particularly given that a large number of clinical trials of novel HER2 protein-anchored drugs are currently ongoing.

## Conclusions

In summary, our data indicate that prognosis of patients with IHC(2+)/FISH(+) tumors do not differ from those with IHC(3+) tumors. The significance of the levels of HER2 protein overexpression in relation to response to anti-HER2 therapies remains unclear. We believe that further investigation is vital to enable provide patients with more personalized treatments.

## Supplementary Information

**Additional file 1. **Clinicopathological features of HER2-positive patients (*n* = 447).

**Additional file 2. **Representative images of HER2 IHC (0, 1+, 2+, and 3+) are shown.

**Additional file 3. **DFS in NAC cases according to HER2 status. Kaplan-Meier analysis indicates disease-free-survival (DFS) according to HER2 status in 133 patients who received NAC containing anti-HER2 drugs. Green and red lines indicate patients with IHC(3+) and IHC(2+)/FISH(+) tumors, respectively.

**Additional file 4. **Patient outcomes stratified by hormone receptor and HER2 status. A-B: DFS (A) and OS (B) are separately analyzed according to hormone receptor (HR) status in all 447 patients. Green and red lines indicate patients with IHC(3+) and IHC(2+)/FISH(+) tumors, respectively. C-D: DFS (C) and OS (D) in the 314 patients who received adjuvant chemotherapies in combination with anti-HER2 drugs. E-F: DFS (E) and OS (F) in the 129 patients who did not receive any chemotherapy.

## Data Availability

The datasets analyzed during the current study are available from the corresponding author on reasonable request.

## Abbreviations

ASCO/CAP: American Society of Clinical Oncology and the College of American Pathologists
DFS: disease-free-survival
EC: epirubicin plus cyclophosphamide
ER: estrogen receptor
FISH: fluorescence in situ hybridization
HER2: Human epidermal growth factor receptor 2
HR: hormone receptor
IHC: immunohistochemistry
NAC: neo-adjuvant chemotherapy
OS: overall survival
pCR: pathological complete response
PgR: progesterone receptor
