# Supplementary material for: Comparison of tumors with HER2 overexpression versus HER2 amplification in HER2-positive breast cancer patients
Source: BMC Cancer. 2022 Mar 5;22:242. doi: 10.1186/s12885-022-09351-4 (PMC8897871; doi:10.1186/s12885-022-09351-4)
Supplement: Supplementary file 1 — Additional file 1. Clinicopathological features of HER2-positive patients (n = 447). [file 12885_2022_9351_MOESM1_ESM.docx]

**Additional file 1. Clinicopathological features of HER2-positive patients (n=447)**

| **Clinicopathological feature** |  | **n^a^** | **%^b^** |
| --- | --- | --- | --- |
| Age (mean, range) |  | 56.4 | 24-88 |
| Tumor size (mm) (mean, range) |  | 17.4 | 0-142 |
| Lymph node metastasis | Positive | 110 | 25 |
|  | Negative | 332 | 74 |
|  | Not evaluated | 5 | 1 |
| Histology | NST | 393 | 88 |
|  | Special type | 54 | 12 |
| Tumor grade^c^ | High | 131 | 29 |
|  | Low/intermediate | 293 | 66 |
|  | Not evaluated | 23 | 5 |
| Ki67 LI (%) (mean, range)^c^ |  | 48.1 | 0-95 |
| ER^c^ | Positive | 288 | 64 |
|  | Negative | 159 | 36 |
| PGR^c^ | Positive | 212 | 47 |
|  | Negative | 235 | 53 |
| HER2^c^ | 3+ | 398 | 89 |
|  | 2+FISH+ | 49 | 11 |
| Chemotherapy | Yes | 318^d^ | 71 |
|  | A+T | 171 |  |
|  | A only | 129 |  |
|  | T only | 18^e^ |  |
|  | No | 129 | 29 |
|  |  |  |  |
| Anti-HER2 therapy | Yes | 328 | 73 |
|  | Tra | 289 |  |
|  | Tra+Per | 38 |  |
|  | No | 119 | 27 |
|  |  |  |  |
| Endocrine therapy | Yes | 277 | 62 |
|  | No | 170 | 38 |

NST: no special type, LI: labelling index, A: anthracycline, T: taxane, Tra: trastuzumab, Per: pertuzumab.

^a^ Presented as n unless otherwise noted to be mean

^b^ Presented as % unless otherwise noted to be range

^c^ Assessed on biopsy for neo-adjuvant chemotherapy (NAC) cases

^d^ Includes 134 patients who received NAC

^e^ Includes one case who received capecitabine as NAC with trastuzumab, but was given taxane after surgery.
