# Supplementary figures and images for: Comparison of tumors with HER2 overexpression versus HER2 amplification in HER2-positive breast cancer patients
Source: BMC Cancer. 2022 Mar 5;22:242. doi: 10.1186/s12885-022-09351-4 (PMC8897871; doi:10.1186/s12885-022-09351-4)

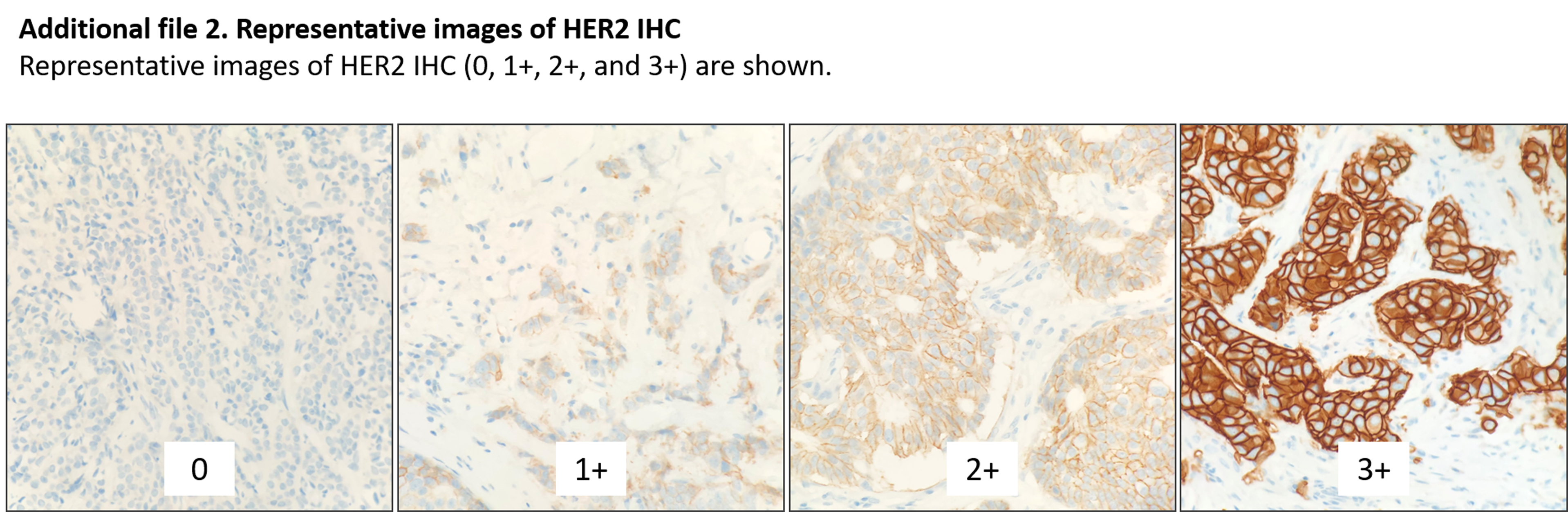

Supplement: Supplementary file 2 — Additional file 2. Representative images of HER2 IHC (0, 1+, 2+, and 3+) are shown. [file 12885_2022_9351_MOESM2_ESM.tif]

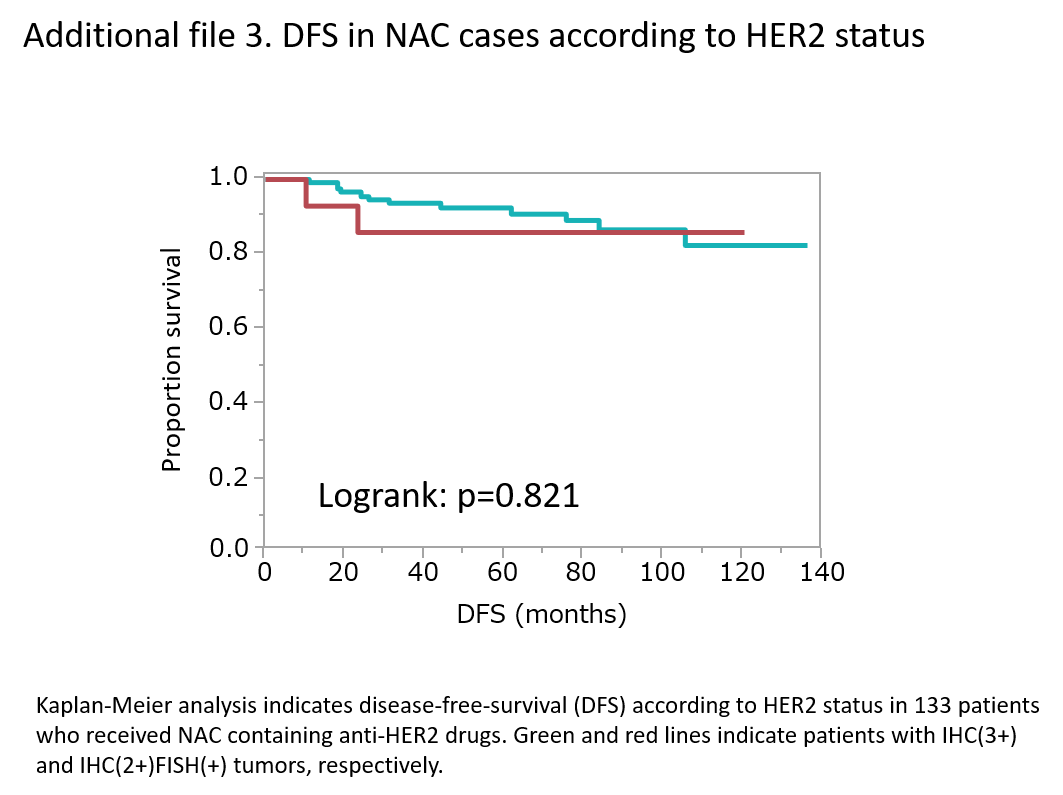

Supplement: Supplementary file 3 — Additional file 3. DFS in NAC cases according to HER2 status. Kaplan-Meier analysis indicates disease-free-survival (DFS) according to HER2 status in 133 patients who received NAC containing anti-HER2 drugs. Green and red lines indicate patients with IHC(3+) and IHC(2+)/FISH(+) tumors, respectively. [file 12885_2022_9351_MOESM3_ESM.tif]
