# Supplementary material for: Comparison of tumors with HER2 overexpression versus HER2 amplification in HER2-positive breast cancer patients
Source: BMC Cancer. 2022 Mar 5;22:242. doi: 10.1186/s12885-022-09351-4 (PMC8897871; doi:10.1186/s12885-022-09351-4)
Supplement: Supplementary file 4 — Additional file 4. Patient outcomes stratified by hormone receptor and HER2 status. A-B: DFS (A) and OS (B) are separately analyzed according to hormone receptor (HR) status in all 447 patients. Green and red lines indicate patients with IHC(3+) and IHC(2+)/FISH(+) tumors, respectively. C-D: DFS (C) and OS (D) in the 314 patients who received adjuvant chemotherapies in combination with anti-HER2 drugs. E-F: DFS (E) and OS (F) in the 129 patients who did not receive any chemotherapy. [file 12885_2022_9351_MOESM4_ESM.pdf]

Additional file 4. Patient outcomes stratified by hormone receptor and HER2 status.
A-B: DFS (A) and OS (B) after stratifying for hormone receptor (HR) status in 447 patients. Green and red lines indicate patients with IHC(3+) and IHC(2+)/FISH(+) tumors, respectively.
C-D: DFS (C) and OS (D) in the 314 patients who received adjuvant chemotherapies in combination with anti-HER2 drugs.
E-F: DFS (E) and OS (F) in the 129 patients who did not receive any chemotherapy.

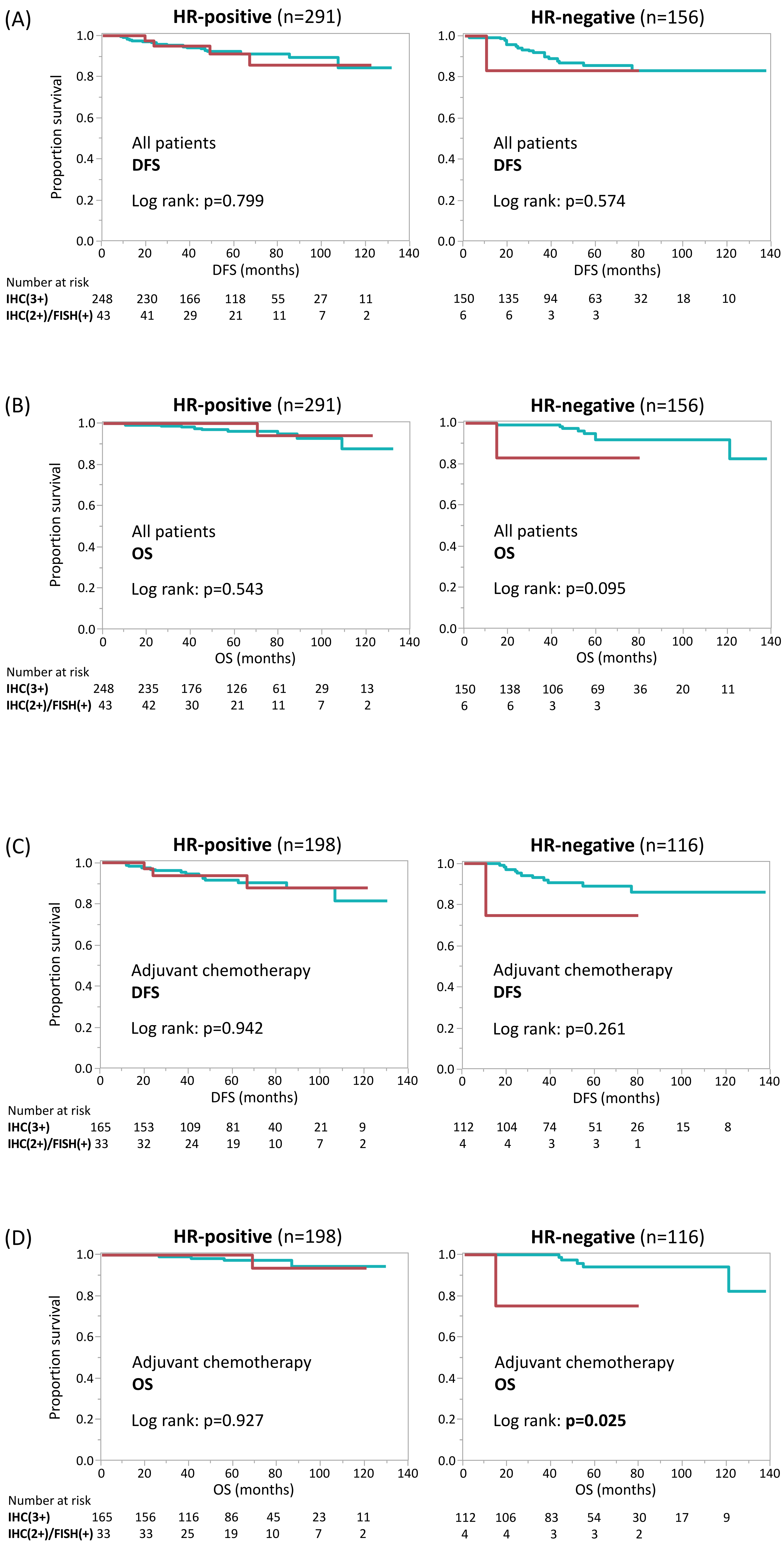

(E)

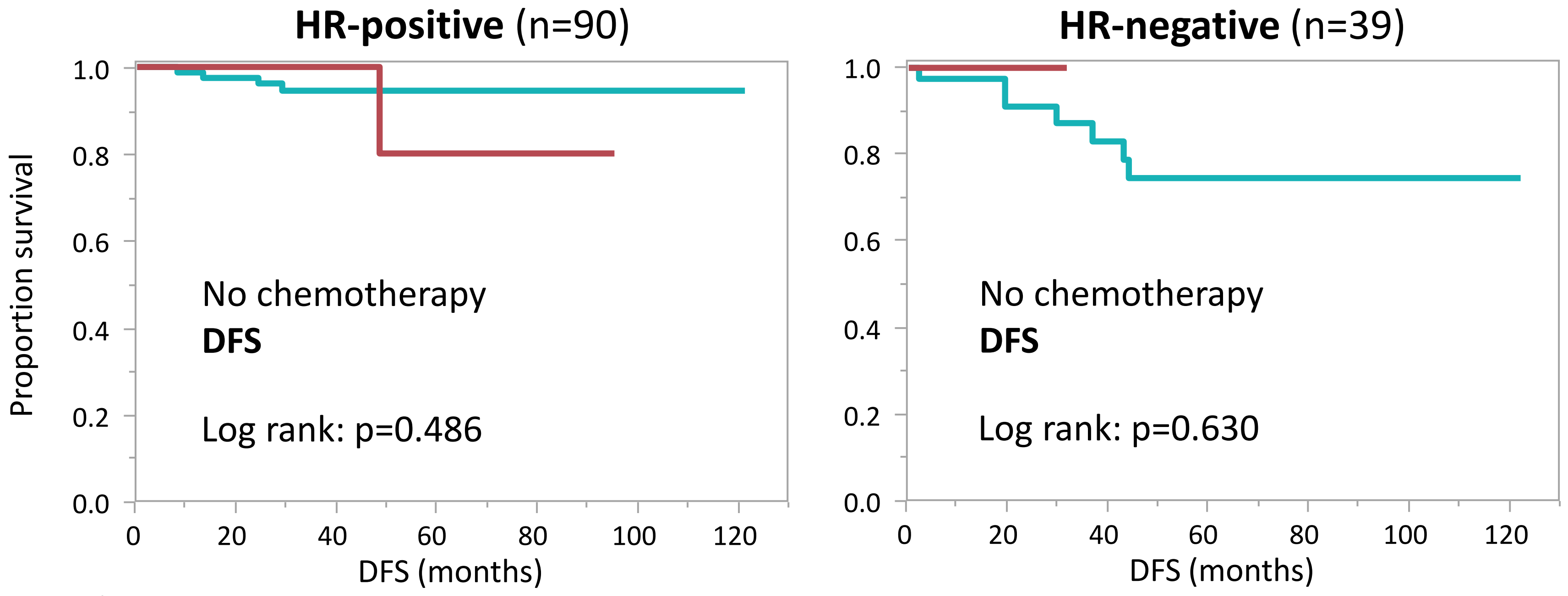

Number at risk

	0	20	40	60	80	100	120
**HR-positive**							
**IHC(3+)**	80	75	56	35	15	9	3
**IHC(2+)/FISH(+)**	10	10	6	3	2		
**HR-negative**							
**IHC(3+)**	37	30	20	11	7	4	2
**IHC(2+)/FISH(+)**	2	2					

(F)

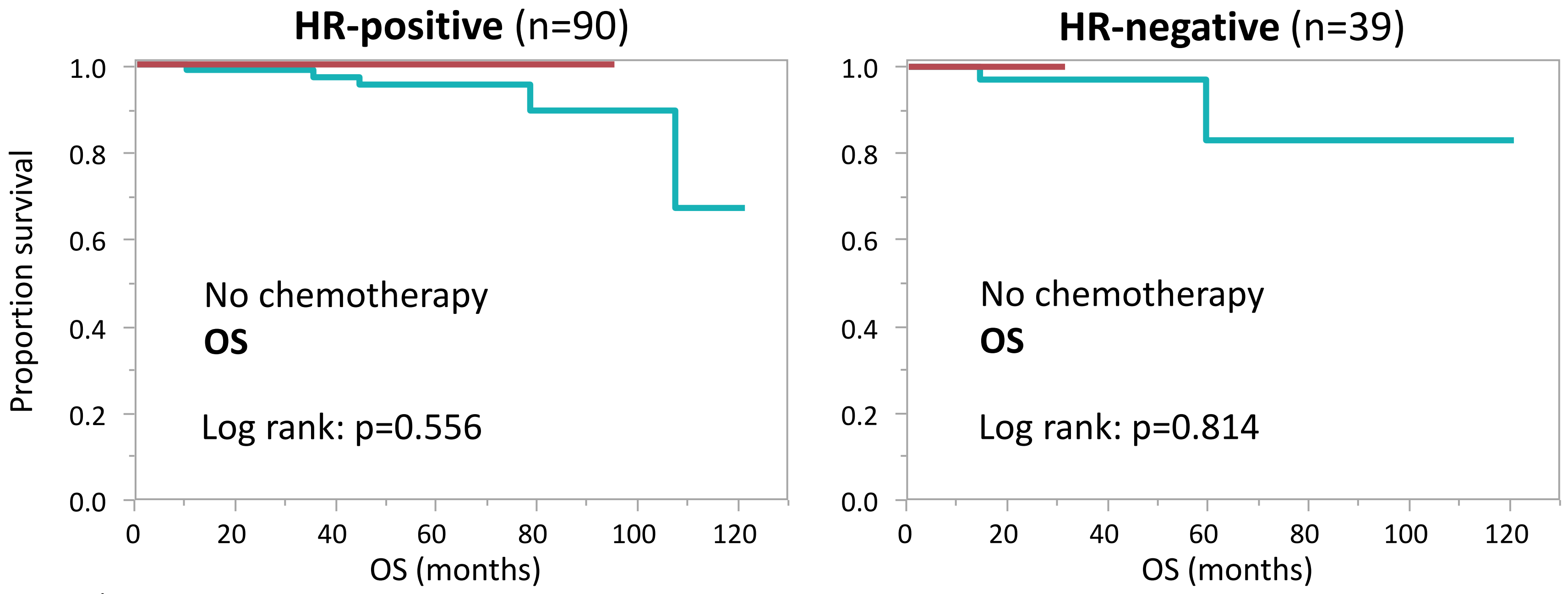

Number at risk

	0	20	40	60	80	100	120
**HR-positive**							
**IHC(3+)**	80	77	59	38	16	9	3
**IHC(2+)/FISH(+)**	10	10	6	3	2		
**HR-negative**							
**IHC(3+)**	37	32	24	14	7	4	2
**IHC(2+)/FISH(+)**	2	2					
